# Heat Stress in Japanese Quails (*Coturnix japonica*): Benefits of Phytase Supplementation

**DOI:** 10.3390/ani14243599

**Published:** 2024-12-13

**Authors:** Apolônio Gomes Ribeiro, Raiane dos Santos Silva, Dayane Albuquerque da Silva, Júlio Cézar dos Santos Nascimento, Lilian Francisco Arantes de Souza, Edijanio Galdino da Silva, José Evangelista Santos Ribeiro, Danila Barreiro Campos, Clara Virgínia Batista de Vasconcelos Alves, Edilson Paes Saraiva, Fernando Guilherme Perazzo Costa, Ricardo Romão Guerra

**Affiliations:** 1 Departamento de Zootecnia, Universidade Federal da Paraíba, Rodovia PB-079, Areia 58397-000, PB, Brazil; raianee.saantos@gmail.com (R.d.S.S.); edilson@cca.ufpb.br (E.P.S.); perazzo63@gmail.com (F.G.P.C.); 2 Departamento de Zootecnia, Universidade Federal Rural de Pernambuco, Rua Dom Manuel de Medeiros, Dois Irmãos, Recife 52171-900, PE, Brazil; dayane.albuquerque.ds@gmail.com (D.A.d.S.); julio.nascimento@ufrpe.br (J.C.d.S.N.); lilian.arantes@ufrpe.br (L.F.A.d.S.); 3 Departamento de Ciências Veterinárias, Universidade Federal da Paraíba, Rodovia PB-079, Areia 58397-000, PB, Brazil; edijanio@veterinario.med.br (E.G.d.S.); danila.campos@academico.ufpb.br (D.B.C.); claravasconcelos@hotmail.com (C.V.B.d.V.A.); 4 Departamento de Gestão e Tecnologia Agroindustrial, Universidade Federal da Paraíba, Rua João Pessoa s/n, Bananeiras 58220-000, PB, Brazil; vange_ribeiro@hotmail.com

**Keywords:** antinutritional factors, calcium absorption, exogenous enzymes, laying quails, phytate

## Abstract

In tropical and subtropical climate regions, heat stress is one of the main causes of production losses in laying quails, aggravated by the antinutritional effects of the phytate in diet ingredients, which negatively affect the bioavailability of minerals, especially calcium and phosphorus. This situation results in a reduction in production and the quality of eggs from commercial laying quails. Several nutritional strategies are utilized to reduce the adverse effects of high temperatures and antinutritional factors such as phytate. Among these strategies, the use of exogenous enzymes, such as phytase, stands out as a viable alternative. Phytase breaks down phytate molecules, optimizing the absorption of essential minerals and improving productive performance and egg quality under unfavorable conditions. Specifically, it is recommended to use 1500 FTU of phytase, as it not only reduces the effects of thermal stress but also enhances eggshell thickness and calcium absorption. In this context, this bibliographic review sought to produce a document demonstrating the beneficial effects of the phytase enzyme on the hydrolysis of the phytate molecule, the availability of calcium for Japanese quails (*Coturnix japonica*), and its implications in thermal stress due to heat.

## Simple Summary

Heat stress in tropical regions significantly affects the production and quality of eggs in laying quails, which is worsened by phytate’s antinutritional effects on calcium and phosphorus absorption. Phytase, an exogenous enzyme, can counteract these effects by breaking down phytate and improving mineral availability. This enzyme’s use in Japanese quails optimizes calcium absorption and enhances their performance under heat stress. Phytase not only improves productivity but also protects the digestive and reproductive systems, promoting bird welfare and sustainability in poultry production.

## 1. Introduction

Japanese quail (*Coturnix japonica*) farming has aroused the interest of breeders due to rapid growth, early sexual maturity, high productivity, low feed intake, and long production period [1,2]. This increased interest also motivates researchers to conduct studies aimed at improving and strengthening quail farming as a highly profitable commercial activity [3].

Exposure of birds to heat stress (HS) environments in tropical and subtropical areas negatively affects their productive performance and causes substantial economic losses [4,5,6]. It is well reported that the poor performance of heat stressed birds is mainly due to reduced feed intake in order to reduce the production of metabolic heat [6,7]. Furthermore, a state of imbalance occurs in the physiology and immunology of birds in response to exposure to HS [8]. In laying quails, exposure to heat of 34 °C decreased egg production and feed conversion, in addition to impairing egg quality [9,10]. Furthermore, yolk and serum cholesterol levels and general stress biomarkers increased in laying quails in heat stress environments [11].

Therefore, many attempts were made to minimize the negative effects of HS on the growth and physiological aspects of birds [12,13], including quails [5,6,9,11,14,15,16]. The use of the phytase enzyme stands out as an alternative, since its administration allows for the effective degradation of the phytate molecule present in diets, resulting in the subsequent release of minerals and other nutrients [17] to be used by animals subjected to heat stress.

Phytase (myo-inositol hexaphosphate phosphohydrolase—IP6) belongs to a class of exogenous enzymes whose function is to degrade the phytate molecule (inositol hexa-phosphate, IP6) present in diet ingredients, releasing phosphorus, calcium, and other nutrients to be used by animals [18,19].

In a study on broiler chickens exposed to heat stress and supplemented with phytase (2000 FTU), Maynard et al. [20] observed improvements in growth performance and a reduction in the incidence of muscular myopathy.

Farias et al. [21] evaluated the thermoregulatory, behavioral, and productive responses of laying hens raised in a hot environment. They reported that hens supplemented with phytase in their diets exhibited improved egg production.

Other studies demonstrated the beneficial effects of using phytase on animal performance [17,22,23], on the quality of the eggs, especially the shells [17,24], and on bone characteristics [25]. It is worth noting that these studies were carried out with broiler chickens and laying hens. In this context, this bibliographic review sought to produce a document demonstrating the beneficial effects of the phytase enzyme on the breakdown of the phytate molecule and the availability of calcium for Japanese quails (*Coturnix japonica*), and the implications on heat stress.

## 2. Japanese Quail (*Coturnix japonica*)

Quails are birds that originated in North Africa, Asia, and Europe. They are members of the Gallinacea order and the Phasianidae family, which also includes chicken and partridge; Pernicidae subfamily, *Coturnix* Genus [26,27]. They are animals that have been bred since ancient times as songbirds, decorative birds, and fighting birds [28]. The first records of domestication date back to the end of the 19th century and beginning of the 20th century [29], when the Japanese began to crossbreed European quails with wild species, resulting in the creation of the domesticated bird known as *Coturnix japonica*, thus marking the beginning of their exploitation [30,31].

In Brazil, the commercial exploitation of quails began around 1989, when a large poultry company decided to set up the first farm in South Brazil and then began exporting frozen quail carcasses [32]. Since then, the activity has become very important in the Brazilian agricultural economy.

There are several attributes that have contributed to the increase in quail production in the country, such as the following: the rapid growth of the birds, precocity in production and sexual maturity (35 to 42 days), high productivity (average of 300 eggs/year), longevity in high production (14 to 18 months), low investment, as well as rapid financial return [26,33], and the need for a small area for production (200–250 cm^2^ bedding system and 150–200 cm^2^ cage system) [34,35].

In recent years, quail egg production has experienced significant growth, driven by increased consumer interest in this product. Eggs are recognized as a complete and balanced food source, rich in protein, and low in fat. Furthermore, their lipid portion contains high concentrations of unsaturated fatty acids [36], giving them a high biological value and making them affordable for consumers [37,38].

For a long time in Brazil and other tropical countries, diets for quails were formulated based on the standards of the National Research Council—NRC [39] and of the Institut National de La Recherche Agronomique—INRA [40], which are based on the nutritional levels established for laying hens and/or broilers. However, this approach often resulted in diets with an excess or deficiency of essential nutrients for quails. A rare exception to this practice is the Nutritional Recommendation Tables for Japanese and European Quails, developed by Silva and Costa [41] based on experiments carried out in Brazil.

The 4th and 5th editions of the Brazilian tables of nutritional requirements for poultry and swine, prepared by Rostagno et al. [42,43], introduced specific requirements for Japanese quails. However, the increasing research on the nutrition of laying quails is of utmost importance. This is due to recent increases in production costs, especially of ingredients used in poultry feed, making it vital to adopt nutritional strategies that maximize nutrient utilization, resulting in greater production efficiency.

## 3. Influence of Heat Stress on Japanese Quail Production

In thermoneutral conditions (range of temperatures where a bird can maintain its body temperature with minimal effort), birds require less energy to maintain a stable body temperature, which allows them to direct most of the energy from food to productive and reproductive processes. Considering the substantial influence of environmental conditions, such as temperature and relative humidity on the production and welfare of birds, it is crucial to monitor and manage these conditions appropriately in order to optimize production [44].

The poultry industry often faces adverse environmental conditions, with exposure to heat stress being one of the most common. Heat stress (HS) occurs when an animal’s heat production exceeds its ability to dissipate it into the environment [45]. In circumstances where the ambient temperature exceeds the thermoneutral range (laying quails, 21 °C to 27 °C [46], birds tend to reduce their physical activity and food intake to control heat production. In addition, they increase panting and water consumption to facilitate heat dissipation through evaporation [47].

Panting is a phenomenon exhibited by birds when they open their beaks to increase the rate of respiration and evaporative cooling of the respiratory tract. During panting, CO_2_ excretion occurs faster than cellular CO_2_ production, which alters the standard bicarbonate buffer system in the blood. The reduction in CO_2_ leads to a decrease in the concentration of carbonic acids (H_2_CO_3_) and hydrogen ions (H^+^). In contrast, the concentration of bicarbonate ions (HCO_3_^−^) increases, thus raising the pH of the blood, i.e., the blood becomes alkaline. To manage this scenario and preserve blood pH balance, birds initiate a process of increased excretion of bicarbonate ions (HCO_3_^−^) and retention of hydrogen ions (H^+^) by the kidneys. Elevated H^+^ alters the acid–base balance, leading to respiratory alkalosis and metabolic acidosis, and it is associated with a decline in the productive performance of birds [48]. However, it is important to note that, although the kidneys play a crucial role in compensating for alkalosis, prolonged exposure to high temperatures can result in the onset of a condition known as chronic alkalosis [49,50].

Furlan et al. [51] conducted a study with five commercial strains of broiler chickens. Subjecting them to heat stress, he observed that, regardless of the strain, there was a decrease in CO_2_ content and an increase in blood pH as the temperature increased [50].

Ruzal et al. [52] state that respiratory alkalosis caused by panting has a deleterious effect on laying hens due to its effect on egg quality. Respiratory alkalosis evidently causes an increase in arterial blood pH associated with a reduction in the partial pressure of CO_2_. This action leads to a decline in plasma bicarbonate levels and an increase in the binding between organic acid and calcium ion content, thus causing declines in the availability of bicarbonate and calcium ions and weakening egg quality.

Laying quails may also suffer impacts on their acid–base balance due to periods of intense heat, resulting in electrolyte and mineral imbalances, potentially culminating in smaller eggs with thinner shells. This phenomenon is mainly attributed to alkalosis, which reduces the availability of free calcium in the blood and increases the proportion of calcium bound to proteins or complexed with organic acids. Consequently, during heat stress, an increase in blood pH is observed due to the loss of carbon dioxide, simultaneously accompanied by a reduction in available calcium [50].

Vercese et al. [10] evaluated the performance and quality of the eggs of Japanese quails subjected to cyclical heat stress and observed that Japanese quails exposed to a temperature of 27 °C already showed signs of heat stress, such as reduced feed intake, egg weight, and egg mass. The cyclical increase in the ambient temperature to 36 °C negatively influenced the percentage of marketable eggs and egg production.

When evaluating the effect of heat stress on laying quails (*Coturnix japonica*), Akdemir et al. [16], stated that birds subjected to temperatures of 34 °C have lower feed intake, lower egg production and weight, in addition to greater feed conversion.

Cruvinel et al. [53], in their evaluation of different electrolyte balance values in the diet of Japanese quails (*Coturnix japonica*), found that heat stress negatively affects bone strength and density, performance, and egg quality.

In their research with laying quails, Moraes et al. [3] investigated the effects of heat stress compared to thermoneutral conditions. They highlighted that exposure to high temperatures can significantly impair bird performance, reflected in reduced feed intake, live weight gain, and efficiency. In addition, they observed a decrease in egg production, as well as in eggshell quality and thickness, due to the lower availability of calcium ions [54].

Lesson and Summers [55] state that, under conditions of respiratory alkalosis, there is a reduction in the calcification of eggs and bones, compromising the performance of birds and contributing to the increased incidence of leg problems in animals, as well as to the production of eggs with thin shells. Alkalosis limits the availability of anions necessary for the formation of calcium carbonate crystals in the eggshell [56] and, consequently, leads to poor egg quality. According to Campos [57], this outcome can result in a 12% reduction in eggshell thickness [10].

Another point is that, in situations of stress due to high temperatures (30–33 °C for quails), there is a decrease in the presence of the calcium transporter calbindin-D28k in the ileum, cecum, colon, and uterus of the birds [3], which results in the deterioration of eggshell quality under these conditions [58]. This occurrence can be explained by heat stress, which reduces the conversion of vitamin D3 into its metabolically active form, 1,25(OH)2D3, essential for the absorption and utilization of calcium [3]. Furthermore, stress also decreases antioxidant patterns, leading to the deficiency of or increased requirements for vitamins and minerals, especially zinc [59], considered a cofactor ion of the enzyme carbonic anhydrase responsible for calcium deposition in the eggshell [19,60].

## 4. Phytase Enzyme and the Hydrolysis of the Phytate Molecule

**Figure 1:**
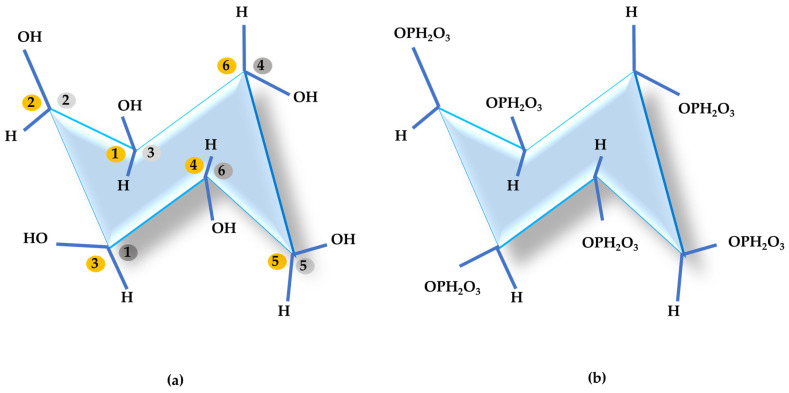
*Myo-inositol* (**a**) and myo-inositol 1,2,3,4,5,6-hexakis dihydrogen phospodium (InsP6) (**b**).

Phytic acid (*myoinositol* 1,2,3,4,5,6-hexakis dihydrogen phospodium) also known as Inositol-6-phosphate (Figure 1), phytate in its salt form [61], phytin, or phytic phosphorus is the main phosphorus storage system in plant seeds, such as cereal grains, nuts, oilseeds, and legumes [61,62]. Table 1 highlights the phytate levels in some foods used in animal feed.

Approximately 60–80% of phosphorus in most cereal grains and oilseeds is bound to phytate [73], which is a chemical form of low availability for poultry and swine [74]. Phytate traps phosphorus and other cationic substances, preventing them from being fully utilized by birds [75]. According to Woyengo and Nyachoti [76], phytic acid contains 12 ionizable protons with pKa values ranging from 1.5 to about 10 [77,78]. Therefore, at all pH values normally observed throughout the gastrointestinal tract, phytate is capable of binding to cations, causing mineral unavailability.

In addition to making phosphorus unavailable, phytate also negatively influences the digestion and absorption of other minerals, amino acids, and the energy utilization of diets [79]. Phytate can create a wide variety of insoluble salts with divalent cations, such as calcium (Ca^2+^), magnesium (Mg^2+^), iron (Fe^2+^), zinc (Zn^2+^), copper (Cu^2+^), and manganese (Mn^2+^) [80], minerals that are considered nutritionally important, especially for laying hens. Thus, blood concentrations of phosphorus and calcium can be reduced, negatively affecting production processes [81]. Furthermore, phytic acid can also bind with proteins and inhibit the activity of some digestive enzymes, including trypsin, pepsin, and alpha-amylase [82].

Phytase or myo-inositol hexaphosphate phosphohydrolase (IP6) is a phosphatase commonly used in the diets of non-ruminant animals as a way to increase the bioavailability of phosphorus phytate and other nutrients [18]. This enzyme hydrolyzes phytic acid and its salts (phytate), producing inositol, inositol monophosphate, and inorganic phosphorus [83], releasing phosphorus, and improving the availability of other nutrients bound to the phytate molecule [84]. The activity of this enzyme is expressed in a Phytase Activity Unit (FTU), which means that the amount of enzyme required to release 1 μmol of inorganic phosphorus per minute of reaction from sodium phytate, at a temperature of 37 °C and pH of 5.5 [85].

**Figure 2:**
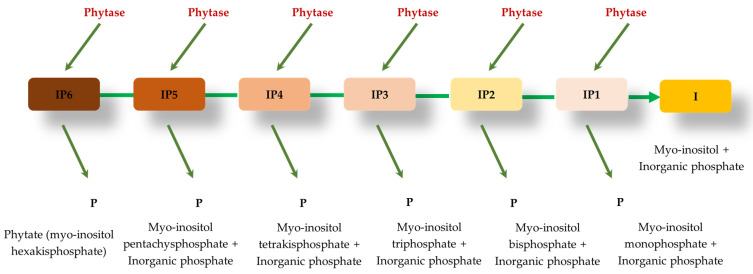
Hydrolysis of the phytate molecule through the action of the phytase enzyme: I (inositol); P (phosphate); IP6 (myo-inositol hexakisphosphate); IP5 (Myo-inositol pentachysphosphate); IP4 (Myo-inositol tetrakisphosphate); IP3 (Myo-inositol triphosphate); IP2 (Myo-inositol bisphosphate); and IP1 (Myo-inositol monophosphate).

Phytase catalyzes the release of phosphate present in phytate and then produces *myo-inositol* pentakis-, tetrakis-, tris-, bis-, and monophosphates, as well as inorganic phosphate [86,87], (Figure 2). Enzymes can be classified into different classes according to some criteria, including the stereospecificity of phytate hydrolysis (carbon number in the myo-inositol ring of phytate at which dephosphorylation is initiated), pH optimum (alkaline or acid phytases), and their catalytic mechanism [88].

**Figure 3:**
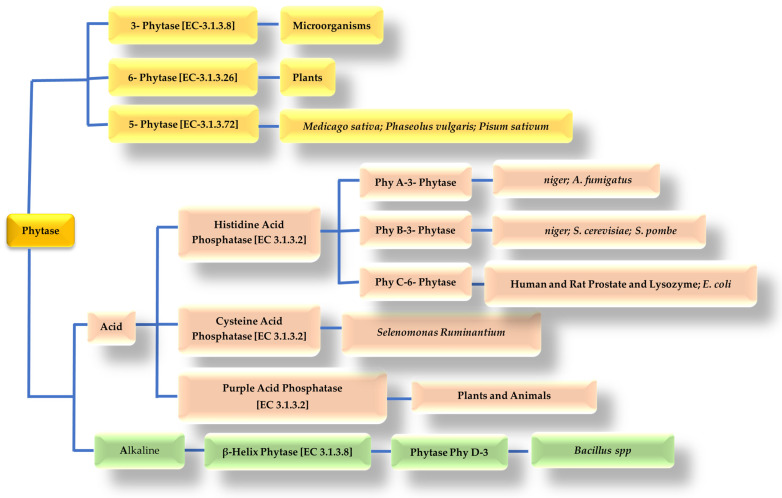
Classification of phytases.

Based on stereospecificity, according to the International Union of Biochemistry (UIB), three groups of phytases can currently be distinguished, depending on the position in the Inositol ring where dephosphorylation is initiated, namely: 3-phytases (EC 3.1.3.8), 6-phytases (EC 3.1.3.26), and 5-phytases (EC 3.1.3.72) [89,90,91], as shown in Figure 3.

Description of the three phytases:

(a) 3-phytase (EC 3.1.3.8): Myo-inositol hexakisphosphate-3-phosphohydrolase, initiates the hydrolysis of the ester bond at the third position of myo-inositolhexakisphosphate into myo-inositol-1,2,4,5,6-pentakisphosphate and orthophosphate, isolated from *Aspergillus niger*, *Neurospora crassa*, *Pseudomonas,* and *Klebsiella* sp. ASR1.

(b) 4-phytase, also known as 6-phytase (EC 3.1.3.26): myo-inositol hexakisphosphate-6-phosphohydrolase hydrolyzes the ester bond at the sixth position of myo-inositolhexakisphosphate to myo-inositol-1,2,3,4,5-pentakisphosphate and orthophosphate, isolated from plants, *E. coli*, *Paramecium*.

(c) 5-phytase (EC 3.1.3.72): Myo-inositol-hexakisphosphate-5-phosphohydrolase, initiates the hydrolysis of the ester bond at the fifth position of myo-inositolhexakisphosphate to myo-inositol-1,2,3,4,6-pentakisphosphate and orthophosphate, isolated from *Medicago sativa*, *Phaseolus vulgaris* and *Pisum sativum*.

Depending on their optimal pH, phytases can be further classified as acidic or alkaline, acidic when their pH is between 2.5 and 6.0, and alkaline when their pH is close to 8.0 [92,93,94]. Based on their structural differences and their catalytic mechanism, phytases can be classified into four groups, which are histidine acid phosphatases (HAPs), ß-helical phytases (BPPs), cysteine phytases (CPs), or purple acid phosphatases (PAPs) [88,95] (Figure 3).

## 5. Phytase Overdose

Phytase overdose refers to the addition of phytase to diets at levels higher than those recommended (500 FTUs/kg) [96] and may even be double or triple these recommendations. Such dosages have been used to effectively combat the antinutritional effects of phytate, improving animal performance [22,84].

According to Cowieson et al. [97], there are three main mechanisms for which the use of high doses of phytase can have beneficial effects:

1. Greater quantity of phosphate made available by the enzyme or greater proportion in the release of calcium and phosphorus;
2. Less phytate excreted, i.e., destruction of the antinutritional effect and increased generation of more soluble esters;
3. Generation of myo-inositol with vitamin/lipotropic effects.

There are several studies demonstrating the relevant effects of the use of phytase overdoses in broiler chicken diets: Nelson et al. [98] used 1–8 g of a phytase from *Aspergillus ficum* with 950 FTUs/kg in the diet of broilers (950 and 7600 FTUs/kg of feed). These authors evaluated that, apparently, 38.9% of the phytate disappeared when using the concentration of 950 FTUs/kg of the enzyme and 94.4% when using 7600 FTUs/kg, with greater responses for weight gain and bone ash in 21-day-old chickens when using 7600 FTUs/kg of phytase in the diets [84,97].

When evaluating the extra-phosphoric effects of overdoses of a microbial phytase produced from *E. coli*, Walk et al. [99] observed better feed conversion in broilers fed with 1500 FTUs/kg of phytase in the diet with a reduction in Ca by 0.16% and P by 0.15%. According to these authors, these findings could be related to the reduction in the antinutritional factors of phytate when using the enzyme in the diet.

When evaluating the effects of phytase overdose (SUNPHASE 5000 G) on the digestibility and bone development of broilers from 1 to 21 days of age, Fernandes et al. [100] observed a quadratic effect of phytase inclusion for the diameter and percentage of Ca and P of the femur, in which the inclusion of 1494 and 1220 FTUs, respectively, presented the best results. For the evaluation of the tibia, the diameter, length, and weight increased linearly with the inclusion of phytase, and, for resistance, a quadratic effect was observed, with 1265 FTUs being the best level.

In a study with four commercially available phytase sources supplemented at regular levels (250 and 500 FTUs/kg) and overdoses (100; 1500 and 2000 FTUs/kg) on live performance, bone mineralization, and apparent ileal digestible energy in broilers from 7 to 24 days of age, Leyva-Jimenez et al. [101] stated that, on days 14 and 22, all phytase sources improved (*p* < 0.05) body weight, weight gain, and bone mineralization when compared to birds on the negative control diet. Overall, phytase supplementation at the S level improved apparent ileal digestibility by 17% at 24 d. Throughout the growing period, the phytase overdose yielded (*p* < 0.05) better performance, bone characteristics, and energy digestibility than at the regular dietary level. In conclusion, all phytase sources were able to compensate for phosphorus deficiency and promote performance and bone mineralization. Higher phytase levels showed a greater response when compared to lower supplementation levels.

Given the satisfactory results with broilers, there is growing interest in the results with laying hens as well, taking into account that the use of Ca and P in laying hens is probably more important than in broilers [102] due to the greater requirement of these minerals in the formation of eggshells [84].

When evaluating the effects of overdose (450 FTUs or 900 FTUs) of two phytases (bacterial phytase or fungal phytase) on the performance, egg quality, biometry of the digestive organs, and bone quality of light laying hens in the first (58 weeks) and second (87 weeks) production cycles, Farias et al. [17] concluded that bacterial phytase produced from Escherichia coli, at a dosage of 450 FTUs, improved the egg production of light laying hens. According to these authors, the use of phytase in the diet of laying hens implies lower feed costs.

When evaluating the effects of an overdose (0; 500; 1000; 1500; and 3000 FTUs) of phytase on bone parameters and the concentration of the epithelial calcium carrier Calbindin-D28k in Japanese quails, Ribeiro et al. [103] had results that showed that phytase overdose not only increased the efficiency of calcium absorption but also stimulated a greater expression of calbindin-D28K in the duodenum and jejunum of the birds. This action resulted in improved calcium mobilization to the tibiae and increased egg production. Additionally, supplementation with 1500 FTUs of phytase reduced the negative effects of heat stress at 36 °C by increasing eggshell thickness. These studies highlight the importance of phytase overdosing as an effective strategy to improve bird health and productivity under heat stress conditions.

## 6. Role of Phytase in Reducing Heat Stress

As already well-documented, heat stress influences the performance of laying hens, as well as in any production system, constituting one of the main causes of production losses in tropical and subtropical regions [104]. Factors such as time of day, genotype, breeding system, and animal density interfere in thermoregulatory responses, leading to changes in the natural behavior of birds [19].

Nutritional strategies are widely used to reduce the negative effects of heat stress, such as adjusting protein and/or energy levels in diets, including fats or oils, supplementing with synthetic amino acids, using vitamins, and offering drinking water with mineral additives [105]. The use of exogenous enzymes such as phytase in the diet of birds stands out as one of these nutritional strategies implemented to overcome the effects caused by heat stress, resulting in better animal welfare [19]. Since birds do not produce enough phytase enzymes to hydrolyze the phytate molecule present in diet ingredients, the introduction of this enzyme in their diets is necessary, as phytase catalyzes the hydrolysis of phytic acid, eliminating its antinutritional properties [82], thus promoting the release of phytic phosphorus present in plant foods, in addition to making other minerals available, such as calcium and vitamins, and increasing the digestibility of the nutrients in diets [21].

Phytase also has extra phosphoric effects, such as the release of proteins, amino acids, carbohydrates, minerals, and vitamins that are complexed to the phytate molecule [106], in addition to improving the utilization of the dietary energy by the animals [101]. The phytase enzyme was also recognized for facilitating the availability of zinc [107]. According to Borges [108], this mineral is incorporated into some dietary formulations to reduce the effects of heat stress in birds, promoting better nutrient absorption during periods of stress, which can reduce losses, especially energy losses.

According to Hu et al. [109], at elevated temperatures, enzyme activity is greatly affected in birds. There is a significant change in the activity of metabolic enzymes in the body, thereby increasing the metabolic rate and therefore increasing the production of free radicals. Excess free radicals disrupt the oxidative and antioxidant balance of the body, leading to lipid peroxidation, DNA and protein damage, and the generation of oxidative stress.

Oxidative damage to the cell membrane by free radicals occurs during zinc deficiency, thus altering the status of enzymes and antioxidant substances [110]. Zinc (Zn) is a vital mineral element found in birds, predominantly in the bones and liver. It plays an essential role in growth and in the proper functioning of the immune system of birds. In addition, zinc contributes to the development of birds, improves feed conversion efficiency, strengthens immunity, and helps prevent diseases [109].

Zinc is required for the activity of over 300 enzymes and participates in many enzymatic and metabolic functions in the body [111]. One of the most important functions of Zn is its participation in the antioxidant defense system. Zinc deficiency increases oxidative damage to cell membranes caused by free radicals [59,111]. Zn exerts its antioxidant action by increasing the synthesis of metallothionein, a protein rich in cysteine, which acts as a free radical scavenger [112]. Another proposed mode of action for Zn as an antioxidant is its interaction with vitamin E, because vitamin E status is impaired in zinc-deficient animals [59]. Furthermore, zinc can occupy iron and copper binding sites in lipids, proteins, and DNA and thus exert a direct antioxidant action [110]. Furthermore, zinc increases antioxidant capacity in birds by increasing the activity of copper–zinc superoxide dismutase and zinc metalloenzymes and altering DNA and the chromatin structure to influence gene expression [109].

Another point related to the importance of the phytase enzyme in the availability of Zn in the diets of birds subjected to heat stress is that this mineral is a cofactor of the enzyme carbonic anhydrase, which plays a vital role in the formation of the eggshell [103]. In addition, Zn can be incorporated in the growth stage during the formation of the calcite crystal [113]. Carbonic anhydrase is an enzyme that catalyzes the hydration of metabolic CO_2_ to HCO_3_^−^, the precursor of eggshell carbonate. Several studies reported that the partial or complete inhibition of carbonic anhydrase can result in thin-shelled or shell-free eggs [113,114]. It was also reported that eggshell quality is reduced by the inhibition of carbonic anhydrase activity or anhydrase mRNA expression in laying hens [113,115,116].

Studies have demonstrated the positive effects of using phytase in the feed of poultry under heat stress: Mohebbifar et al. [117] investigated the effects of phytase supplementation (0 and 150 FTUs) in diets with different levels of rice bran and non-phytic phosphorus on productive performance, egg quality, leukocyte profile, and serum lipids in laying hens raised under high environmental temperatures. They found that diets with high phytate content (such as those rich in rice bran) and/or low non-phytic phosphorus (2.5 g/kg), when supplemented with phytase, resulted in significant improvements in the performance and egg quality of birds subjected to heat stress.

When evaluating the simple and combined effects of phytase and citric acid on performance, nutrient digestibility, bone characteristics, intestinal morphology, and blood parameters of Japanese quails (aged 1 to 35 days) and fed with low-phosphorus diets, Hezaveh et al. [118] found that phytase supplementation resulted in significant improvements. There was an increase in bird performance, protein, ash and phosphorus digestibility coefficients, metabolizable energy, bone mineralization, and intestinal morphology, even with a 0.12% reduction in non-phytic phosphorus in the diet.

When evaluating phytase levels (0; 200; 400 and 600 FTUs/kg) in the diet on the performance and quality of eggs of Japanese quails subjected to heat stress, Lima et al. [119] concluded that the use of phytase improved the performance and quality of the eggs of the birds. The best phytase level for better efficiency in the use of phosphorus by the birds and for better egg mass was 463 FTUs/kg.

When evaluating the effects of an overdose of phytase on bone parameters and the concentration of the epithelial calcium carrier Calbindin-D28k in Japanese quails under heat stress, Ribeiro et al. [103] demonstrated that phytase supplementation brought benefits to the birds, especially those kept at temperatures of 30 °C, given the greater demand for vitamins and minerals, due to changes in their metabolism. Phytase improved Ca absorption efficiency and positively influenced the increased expression of anti-calbindin-D28K in the duodenum and jejunum of the birds. This action provided greater Ca mobilization to the tibiae and higher total egg production for the birds kept at this temperature. Furthermore, these results demonstrate that dietary phytase supplementation at a level of 1500 FTUs was able to reduce the deleterious effects of heat stress (36 °C) and positively influenced the increase in quail eggshell thickness.

In this context, it can be seen that the use of phytase can positively influence the reduction in the effects of heat stress and antinutritional factors such as phytate in Japanese quails.

## 7. Calcium and Phosphorus in the Diet of Laying Quails (*Coturnix japonica*)

According to current practice, diets for commercial laying quails are formulated based on the recommendations of tables of nutritional requirements specific to each lineage, such as editions 4 and 5 of the Brazilian Tables of Nutritional Requirements for Poultry and Swine, prepared by Rostagno et al. [42,43], National Research Council [39], and the Agriculture and Food Research Council (AFRC) [120]. Reducing the density of nutrients such as phosphorus in the feed and using phytase supplementation can reduce the total cost of the diet, thus contributing to the profitability of egg production [121].

Calcium (Ca) and phosphorus (P) are essential for laying quails, and their availability is most crucial during the laying period [122]. Among all the minerals, calcium and phosphorus play the most fundamental role in the construction of the skeleton, from 80 to 85% of its structure. These minerals are essential in the formation of eggshells and in muscle development. Thus, they are indispensable for the proper functioning of the animal’s body [123].

Calcium is the most abundant mineral in the animal organism, representing a body proportion of 1:75 [124]. Most calcium (99%) is present in bones [125,126] in the form of hydroxyapatite, an inorganic crystalline structure composed of calcium and phosphorus [Ca_10_(PO_4_)_6_(OH)_2_] [124]. During the processes of absorption, metabolism, and excretion, calcium and phosphorus interact, maintaining a ratio of approximately 2:1 [127]. In addition to composing the bone structure, calcium is also present in soft tissues (1%), where it performs several functions, such as activating enzymes, transmitting nerve stimuli, and participating in the blood coagulation process [124].

The use of calcium by the bird’s body varies mainly with age. During the growth period of laying hens, most of the dietary calcium is directed towards the formation of bones [124]. In contrast, in the adult phase, calcium is predominantly used for egg production, specifically for shell formation [123]. The Ca necessary for the formation of the shell, which comes from bone resorption and intestinal absorption, is transported by the blood to the calcigenous chamber [124,128]. This transport is mediated by the activity of carbonic anhydrase, a crucial enzyme in eggshell formation [113].

Approximately 30% of the calcium required for eggshell formation is mobilized from the bones. This rate is because, in the uterus, where calcium carbonate is deposited, there is no calcium storage. The organic fraction of the shell is synthesized by glands, and calcium is mobilized directly from the blood, and the transfer of plasma to the uterus occurs very quickly [124,129].

The most obvious symptoms of calcium deficiency in young birds include delayed growth, reduced feed intake, and bone fragility [124]. In adult birds, the deficiency manifests itself in reduced egg production, thin-shelled eggs, and reduced ash and calcium content in bones [130,131].

Phosphorus, in turn, plays a crucial role in both the absorption and metabolism of calcium [132]. More than 80% of phosphorus is associated with calcium in bone formation, while the remainder is present in soft tissues [133,134]. Its functions include participation in bone structure (hydroxyapatite), in cell membranes in the form of phospholipids (lecithin), as phosphate in deoxyribonucleic acid (DNA) and ribonucleic acid (RNA) molecules, and in the form of energy molecules, such as adenosine diphosphate (ADP) and triphosphate (ATP) [132,133].

The skeleton functions as a reservoir of calcium and phosphorus in the body, and this function is especially crucial in laying hens, since the eggshell contains approximately 10% of the total calcium in the bird’s body [124]. Therefore, there is a high demand for calcium to maintain blood homeostasis while the shell is forming [135]. Physiological mechanisms regulate skeletal integrity in response to different needs during the productive life of laying hens. The relative importance of the intestine and bones as sources of calcium depends on the concentration of this mineral in the diet [124,136].

In general, there are intestinal barriers, such as physical–chemical conditions, pH, and viscosity, which make it difficult to absorb most minerals [137]. Therefore, dietary levels of calcium and phosphorus are often higher than actual requirements, resulting in a lower utilization rate and environmental impact due to excess excretion [138].

To ensure eggshell quality, calcium and phosphorus levels in the diet must meet the nutritional requirements of the birds [139]. It is important to note that these requirements may vary depending on the type of quail (Japanese or European), sex, age, and purpose of the farm, whether for the production of eggs for consumption, incubation, or for meat [140].

Several studies are being conducted to establish precise information on the nutritional needs of Japanese quails, aiming to achieve maximum productive performance and maintain health. Table 2 presents the recommended values of calcium and available phosphorus, proposed by different authors for Japanese quails in the laying phase.

Variations in calcium (Ca) and available phosphorus (P) recommendations for Japanese quails derive from several reasons, such as the source and bioavailability of the minerals, the age of the birds, the efficiency of absorption, and the energy levels of the diets.

## 8. Calcium (Ca) Absorption

Active absorption and reabsorption of Ca is of great importance in maintaining the homeostasis of this mineral for many physiological functions [152,153]. It is also essential for the formation of skeletal hydroxyapatite and for the mineralization of bones, as well as for satisfying the high Ca requirement in the uterus (where the eggshell is formed) for egg calcification in laying quails [154,155].

In modern commercial strains of laying quails, about 2–3 g of Ca (equivalent to 10% of total body calcium) is transferred daily for deposition in the eggshell [122,156]. The largest source of Ca comes from dietary absorption in the intestine, renal reabsorption, and bone stores [152].

Plasma Ca concentrations are precisely controlled within a narrow range of variation, both intracellular and extracellular, through interactive mechanisms between the parathyroid hormone (PTH), calcitonin (CT), 1,25 dihydroxycholecalciferol (1,25(OH)2D3) and estrogen, and their respective receptors located in the intestine, bones, and kidneys [124]. In response to low blood Ca^2+^ levels, parathyroid cells rapidly release PTH, which stimulates bone resorption by releasing calcium from the bone matrix into the circulation and also by promoting reabsorption in the kidneys. These actions help restore serum Ca^2+^ concentrations. In chronic situations, PTH increases the production of 1,25(OH)2D3 to facilitate intestinal Ca^2+^ absorption Ca^2+^ [157].

Among the various mechanisms aimed at reducing circulating Ca, phytate can be highlighted as one of these, as it has chelating power over several divalent cations, mainly Ca, making it less available [81,158].

There are two mechanisms for the mobilization of Ca that is deposited in the bones. The first is represented by the transfer of ions from the hydroxyapatite crystals to the interstitial fluid, from which the calcium passes into the blood. This mechanism is considered purely physical and takes place mainly in the spongy bones. The second mechanism, which is characterized by having a slower action on the bone tissue, uses the parathyroid hormone (PTH) produced in the parathyroid gland [159]. The PTH increases the number of osteoclasts and the resorption of bone matrix [160], releasing calcium phosphate and increasing calcemia [161] and acts on the kidneys, decreasing phosphorus excretion and stimulating the synthesis of active vitamin D [162]. Parathyroid hormone secretion activity is regulated in response to fluctuations in calcium concentration [124,163].

Calcitonin is another hormone that acts on Ca metabolism to maintain normal levels in blood plasma. It is produced by the parafollicular cells of the thyroid and acts by inhibiting the reabsorption of bone matrix and, consequently, the mobilization of bone Ca. Its secretion is stimulated when Ca levels are high in the blood [164,165].

Modern commercial strains of layers can produce about 300 eggs per year, which require an amount of Ca equivalent to more than 20× their total body weight annually [166,167]. Therefore, layers must absorb a large amount of calcium to support the demand for this production. Epithelial calcium absorption involves two distinct pathways: active transcellular transport of calcium and diffusion via the paracellular pathway [152].

In the mammalian intestine, transcellular calcium transport plays an important role in calcium homeostasis, which is a three-step process comprising the passive entry of calcium into the enterocyte via TRPV6; the cytoplasmic transfer of calcium bound to the protein calbindin-D9k; and, in birds, the analogous protein is calbindin-D28k; and calcium extrusion across the basolateral membrane via Ca^2+^-ATPase (PMCA 1b) and/or Na–Ca exchanger [168,169]. A similar mechanism exists in tissues such as the kidney, uterus, and placenta [152,170,171,172,173,174,175].

The kidney also plays an essential role in calcium balance by regulating the excretion of calcium from the body. To meet the requirement for eggshell calcification, calcium filtered in the glomerulus is extensively reabsorbed as it passes through the nephrons [172,176].

Egg formation in laying quails is a complex process, which depends on the maturation of the ovum in the ovary, the passage of the mature oocyte through the oviduct via the infundibulum, the addition of albumen in the magnum, the synthesis of the membrane in the isthmus, and deposition of the shell in the uterus. The mineral components of the shell in laying quails are composed mainly of calcium carbonate and, to a lesser extent, of magnesium carbonate and tricalcium phosphate. The initial deposition of minerals occurs in the isthmus where the shell membrane is formed. The calcification of the shell in the uterus takes approximately 20 h, during which time 2 g of Ca is deposited [177].

The function of the different cell types of the uterus and tubular glands is not clearly defined, but they are implicated in the process of Ca secretion. High concentrations of calcium-activated ATPase is located mainly in the microvilli of the uterus tubular cell glands [178]. Although the pathway of calcium absorption and reabsorption is physiologically known, there are no studies in quails that aim to elucidate the absorption, reabsorption, and deposition of calcium in the uterus, including its sites and expression, and they are also scarce in poultry farming in general.

## 9. Transepithelial Calcium Transport Mediated by TRPV6 and Calbindin-D28K

Epithelia play a fundamental role as linings in various biological compartments, where they create specialized surfaces for essential functions such as protection, secretion, absorption, and reabsorption of substances [179]. For example, the ability of epithelial cells to regulate the absorption and secretion of vital ions, such as calcium, plays a crucial role in maintaining electrolyte balance, thus ensuring the proper processes of several vital functions in the body [172,180,181].

**Figure 4:**
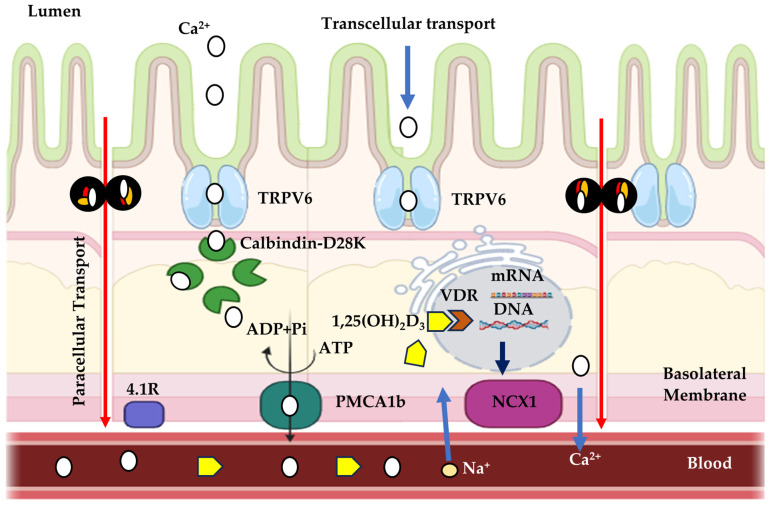
Schematic drawing of transepithelial calcium transport. Integrated model of active epithelial Ca^2+^ transport. Ca^2+^ enters the cell from the luminal side via TRPV6, subsequently binds to calbindin-D28K, and is extruded at the basolateral membrane by a Na+/Ca^2+^ exchanger (NCX1) and/or a plasma membrane Ca^2+^-ATPase (PMCA1b). The active form of vitamin D [1,25(OH)2D3] stimulates the individual steps of transcellular Ca^2+^ transport by increasing the expression level of TRPV6, calbindin-D28K and the extrusion systems. TRPV6 (transient receptor potential vanilloid channel type 6); VDR (vitamin D receptor).

The absorption and reabsorption of calcium in the body occurs through the epithelium of various organs, such as the intestines, kidneys, mammary glands, and placenta. This process is mediated by a complex sequence of events regulated by different factors, such as pH, extracellular calcium concentrations and hormones [172,182]. There are two main pathways described for calcium transport across epithelia: the paracellular pathway, which allows the movement of ions through the spaces between cells, and the transcellular pathway, in which ions are transported through the cytoplasm of epithelial cells. In the transcellular pathway, calcium transport occurs through a process that involves several steps, including the entry of calcium into the cell through the apical membrane, the translocation of calcium through the cytoplasm to the basolateral membrane, and, finally, the release of calcium into the blood [172] (Figure 4).

Calcium entry into epithelial cells occurs through channels located in the apical membrane, known as TRPV5 and TRPV6 (Transient Receptor Potential Vanilloid), previously called ECaC1 and ECaC2, respectively. These proteins belong to the family of channels known as Transient Receptor Potential Vanilloids, specifically grouped in the Vanilloid subfamily [172], which includes six components, with only TRPV subtypes 5 and 6 having calcium transport capacity [183].

**Figure 5:**
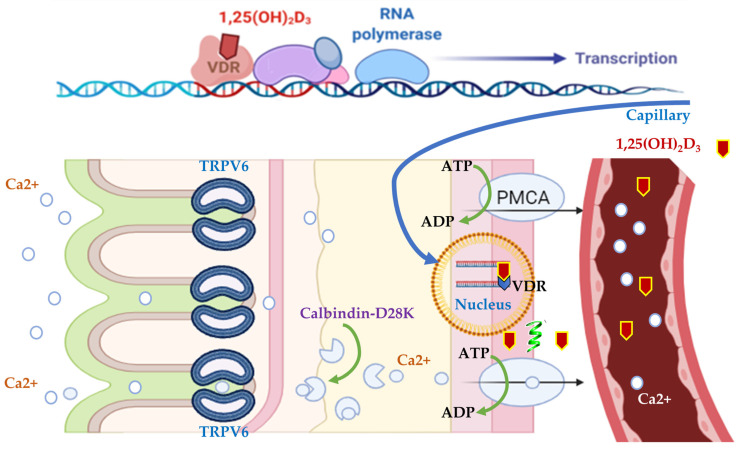
Calcium selective channel TRPV6 and Calbindin-D28K. TRPV6 (transient receptor potential vanilloid channel type 6); VDR (vitamin D receptor).

The ion channel TRPV6 (Transient Receptor Potential Vanilloid Channel Type 6) (Figure 5) functions as an epithelial Ca channel in the intestine, kidney, bone, skin, placenta, and exocrine glands of mammals, which are tissues that are characterized by a high demand for calcium transport [172,184].

TRPV6 is described as having a facilitating effect on Ca entry into epithelial cells, with a significant correlation between TRPV6 expression and Ca transport [154,185], and this ion channel is vitamin D dependent [186]. Observations have led to the suggestion that TRPV6 is an important limiter of calcium entry and homeostasis [154,187]. This ion channel is expressed in the absorption and reabsorption epithelia, that is, in the intestine and kidney; however, there is little information about the expression pattern of TRPV6 in laying hens [152] and none for laying quails.

Immunohistochemical studies indicated anti-TRPV6 positivity in the apical border of the cells of the duodenum, jejunum, ileum, cecum, and rectum. The positivity was highest in the duodenum and weakest in the rectum and was absent in the crypts and goblet cells. PCR and Western blot also demonstrated the presence of TRPV6 in the aforementioned segments, mainly in the duodenum and jejunum of laying hens [152]. This finding is consistent, since the main sites of calcium absorption in birds are the duodenum and jejunum [188]. In humans, TRPV6 (mRNA) is also most abundant in the duodenum and jejunum, with low levels in the ascending colon, and none in the ileum or distal segments of the large intestine [189].

The significant presence of TRPV6 (mRNA and protein) in the ileum of laying hens [152], contrasts with that found in rats, humans, and sheep [184,189,190], suggesting that the ileum may also have a significant role in intestinal absorption in laying hens. In the kidney of laying hens, TRPV6 (protein) is positive at the apical border of the proximal convoluted tubules, loops of Henle, and distal convoluted tubules. However, it is weaker in the digestive system.

Despite the cited study [152], for other authors [191,192,193], the presence of TRPV6 in bird tissues, including laying hens or quails, is still uncertain. Considering that TRPV6 is found in the intestinal segments of laying hens, calcium absorption should play a significant role in egg production during laying peak, including shell production, in the uterus. These observations are in line with Yang et al. [152], who describe areas for future studies covering the mechanisms of calcium transfer in laying hens. These studies would include the expression pattern of TRPV6 between laying and non-laying hens, co-expression with other vitamin-dependent proteins such as calbindin-D28k, and expression in other calcium-transporting tissues such as the avian uterus and bone marrow.

Thus, Yang et al. [152] propose that the epithelial channel TRPV6 has a crucial function in the absorption and reabsorption of calcium in laying hens. However, further studies are needed to elucidate its action, especially in the uterus and kidney, and there are still no studies of such calcium transporters in quails. Thus, the increase in the expression and positivity of TRPV6 may physiologically prove the better use of calcium after environmental or nutritional changes.

The Calbindin-D28K protein (Figure 5) plays a crucial role in calcium transport: once in the cytoplasm, calcium ions bind with high affinity to calbindin D28K, which acts as a carrier facilitating the diffusion of calcium between the apical and basolateral cytoplasm of cells [3].

Passive calcium absorption involves the diffusion of calcium from the intestinal lumen into the enterocyte, while active absorption is the process in which calcium passes through channels found in the apical membrane of the microvilli of the enterocytes. These then bind to a protein that binds to calcium, Calbindin (vitamin D-induced calcium-binding protein), and diffuses it into the cytoplasm, which is finally extruded by CA^2+^-ATPase in the basolateral membrane, reaching the vascular system through vessels of the lamina propria [156].

Thus, the potential for active calcium absorption depends on the presence of calbindin in the cytoplasm [194], with its biosynthesis being dependent on circulating levels of vitamin D [195], and on the activity of Ca^2+^-ATPase in the basolateral membrane [196], with the majority of dietary calcium being absorbed by the intestinal segments via the active pathway [197].

Calbindin exists in two main forms, with low molecular weight, one 9kDa protein (Calbindin-D9k) present in mammalian intestines, and another with high molecular weight with 28kDa (Calbindin-D28k), present in the kidney, brain, intestine, and uterus of birds, and in the kidneys of mammals [198].

The localization of calbindin-D28k in birds was initially described in the intestine of chickens [191] and later in the uterus of laying hens [199] and in the distal portion of the isthmus [200], the latter locations being sites of calcium deposition in the avian oviduct. The synthesis of calbindin-D28k is dependent on vitamin D [199]. However, during the eggshell production cycle, the concentration of calbindin-D28k in the uterus is relatively insensitive to changes in plasma vitamin D levels [201,202].

In the intestine of laying hens, there is positivity (protein) of calbindin-D28k in all segments. It is higher in the duodenum and jejunum, mainly in the apical portions of the villi [156,195], and lower in the ileum. Positivity also occurs in the cecum and colon, to a lesser extent, which indicates that laying hens absorb calcium in both the small and large intestines [156]. Intestinal calbindin-D28k mRNA expression in laying hens is twice as high in laying hens (25 weeks) than in immature hens (11–17 weeks). However, these immature hens do not show positivity (protein) for anti-calbindin-D28k [195]. Despite the mode of action of calbindin-D28k demonstrated in the aforementioned study in laying hens, it is still not known how it behaves in other laying species such as quails, and the raising of quails has been growing in the northeast region of Brazil [30].

It is well established that there is an increased requirement for dietary calcium after sexual maturation in laying hens. This increase is reflected in the elevated expression of calbindin-D28k in the enterocytes of laying hens when there is egg production [195]. The concentration of calbindin-D28k would also be related to the amount of calcium transported to the eggshell in the uterus of laying hens [203], and these levels would be induced by the increase in estrogen. Therefore, calbindin-D28k would modulate the intestinal absorption capacity of calcium [3] and its deposition in the uterus [103,203], influencing the production and quality of the eggshell.

It is known that stress due to high temperatures (30–33 °C) decreases the presence of calbindin-D28k in the ileum, cecum, colon, and uterus of birds, causing the deterioration of eggshell quality under this condition [58]. This finding can be explained by the fact that heat stress reduces the conversion of vitamin D3 into its metabolically active form, 1,25(OH)2D3, which is essential for the absorption and utilization of calcium [103]. In fact, the calcium requirement for laying hens increases under conditions of high environmental temperatures [3]. Therefore, the increase in the expression of and positivity to Calbindin-D28k can also physiologically prove the better use of calcium after a specific diet or environmental treatment.

## 10. Conclusions

The incorporation of phytase into Japanese quail diets is a promising strategy to optimize poultry production. The ability of the phytase enzyme to release essential nutrients is crucial to improving performance and egg production, allowing quails to reach their maximum potential. In addition, phytases help to reduce the negative effects of heat stress on the functions of epithelial calcium transporters, preserving the digestive and reproductive integrity of laying quails. This ability to counteract the impacts of heat stress further highlights the benefits of phytases in poultry production, contributing to the sustainability and quality of production, in addition to promoting bird welfare. In summary, the addition of phytase to quail diets is an effective and comprehensive approach to increase production efficiency. It is recommended to use a concentration of 1500 FTUs of phytase, as this dosage not only reduces the effects of thermal stress but also improves eggshell thickness and calcium absorption, contributing to maximizing the productive performance of quails. Future research should focus on exploring the synergistic effects of phytase in combination with other nutritional strategies, and how these approaches might further enhance heat stress tolerance in quails.

## Figures and Tables

**Table 1:** Phytate content in foods (based on dry matter).

Products	Phytate, %	Reference
Corn	0.78–1.05	[63,64]
Soybean	1.01–1.47	[64,65]
Sorghum	0.80	[66]
Cottonseed meal	2.65	[66]
Corn germ	2.97	[66]
Polished rice	0.60	[64,67]
Oats	0.79–1.01	[64,65]
Wheat	0.39–1.35	[64,68]
Soybean bran	1.0–1.5	[69]
Rice bran	5.90–6.48	[70,71]
Wheat bran	5.38	[63,64]
Whole wheat flour	2.22	[64,72]
White wheat flour	0.404	[64,72]

**Table 2:** Recommendations for available calcium (Ca) and phosphorus (P) for Japanese quails.

Age—Weeks	Ca (%)	P (%)	Literature
6–16	3.50	0.45	[141]
6–29	2.00–3.05	-	[142]
6–19	3.51	-	[143]
8–21	2.50	0.31	[144]
45–57	3.50	0.15	[145]
45–57	3.80	-	[146]
7–54	2.50	0.25	[139]
12–42	2.50	0.35	[147]
26–38	2.00	0.31	[148]
21–36	3.10	0.32	[149]
7–20	2.50	0.35	[150]
8–56	2.99	0.31	[42]
6–57	2.0–3.8	0.15–0.45	[124]
20–32	-	0.39–0.44	[151]
9–24	2.68	0.38	[123]
8–56	3.01–3.04	0.31–0.80	[43]

## Data Availability

The data that support the findings of this study are available on request from the corresponding author.
